# Influenza a virus triggers acute exacerbation of chronic obstructive pulmonary disease by increasing proinflammatory cytokines secretion via NLRP3 inflammasome activation

**DOI:** 10.1186/s12950-022-00305-y

**Published:** 2022-06-23

**Authors:** Shuang Ji, Meng-Yuan Dai, Yun Huang, Xiang-Chun Ren, Meng-Long Jiang, Jin-Ping Qiao, Wen-Ying Zhang, Yuan-Hong Xu, Ji-Long Shen, Ren-Quan Zhang, Guang-He Fei

**Affiliations:** 1 Department of Respiratory and Critical Care Medicine, First Affiliated Hospital of Anhui Medical University, Hefei, Anhui China; 2 Key Laboratory of Respiratory Disease Research and Medical Transformation of Anhui Province, Hefei, Anhui China; 3 Department of Thoracic Surgery, First Affiliated Hospital of Anhui Medical University, Hefei, Anhui China; 4 Department of Clinical Laboratory, First Affiliated Hospital of Anhui Medical University, Hefei, Anhui China; 5 Department of Pathogen Biology and Provincial Laboratories of Pathogen Biology and Zoonoses, Anhui Medical University, Hefei, Anhui China; 6 Department of Respiratory and Critical Care Medicine, Fourth Affiliated Hospital of Anhui Medical University, Hefei, China

**Keywords:** Airway inflammation, Acute exacerbation, Chronic obstructive pulmonary disease, Influenza A virus, MCC950, NLRP3 inflammasome pathway

## Abstract

**Background:**

Influenza A virus (IAV) triggers acute exacerbation of chronic obstructive pulmonary disease (AECOPD), but the molecular mechanisms remain unclear. In this study, we investigated the role of IAV induced NLRP3 inflammasome activation to increase airway inflammation response in the progression of AECOPD.

**Methods:**

Human bronchial epithelial cells were isolated and cultured from normal and COPD bronchial tissues and co-cultured with IAV. The NLRP3 inflammasome associated genes were identified using RNA sequencing, and the expressions of NLRP3 inflammasome components were measured using qRT-PCR and western blot after cells were transfected with siRNA and treated with MCC950. Moreover, IAV-induced COPD rat models were established to confirm the results; 37 AECOPD patients were included to measure the serum and bronchoalveolar lavage fluid (BALF) of interleukin (IL)-18 and IL-1β.

**Results:**

Increased levels of NLRP3 inflammasome components were not seen until 6 h post-inoculation in normal cells. However, both cell groups reached peak NLRP3 level at 12 h post-inoculation and maintained it for up to 24 h. ASC, Caspase-1, IL-1β and IL-18 were also elevated in a similar time-dependent pattern in both cell groups. The mRNA and protein expression of the NLRP3 inflammasome components were decreased when COPD cells treated with siRNA and MCC950. In COPD rats, the NLRP3 inflammasome components were elevated by IAV. MCC950 alleviated lung damage, improved survival time, and reduced NLRP3 inflammasome components expression in COPD rats. Additionally, the serum and BALF levels of IL-1β and IL-18 were increased in AECOPD patients.

**Conclusions:**

NLRP3 inflammasome is activated in COPD patients as a pre-existing condition that is further exacerbated by IAV infection.

**Supplementary Information:**

The online version contains supplementary material available at 10.1186/s12950-022-00305-y.

## Background

Chronic obstructive pulmonary disease (COPD), the fourth cause of chronic morbidity and mortality worldwide, is characterized by chronic airway inflammation [1, 2]. Acute exacerbation of AECOPD is regarded as an infective event due to its involvement in viral or bacterial respiratory tract infections [3, 4]. Influenza A viruses (IAVs) are common respiratory pathogens in humans. Notable features of these infections are exaggerated cellular influx into the lungs and elevated concentrations of inflammatory mediators linked to hypercytokinemia or ‘cytokine storm’, which is indicative of severe symptoms and poor disease outcome [5]. Consequently, understanding the mechanisms involved in cytokine storm development is imperative to the creation of improved and better-targeted treatments to reduce mortality associated with IAV. Our previous studies have shown that IAV was the most common triggering agents in AECOPD patients [6]. Studies performed by other groups revealed that patients with COPD are more susceptible to influenza and suffer more severe symptoms [7]. Nevertheless, the underlying cellular and molecular mechanisms of airway inflammation activated by IAV in AECOPD are poorly understood.

Based on antigenic differences, influenza virus is broadly classified into three antigenic types: A, B and C. Only two IAV subtypes (i.e., H1N1 and H3N2) are currently in general circulation, and seasonal influenza A virus (H3N2) is more common. Over the last several years, IAV/H3N2 strain have accounted for most influenza A infections and is the most common cause of influenza-related hospitalizations and deaths in adults with chronic disease [8]. Therefore, we selected the H3N2 strains as an experimental candidate to explore the underlying mechanisms of influenza A infection. H3N2 is not only the most common strain, but also its shares the similar mechanisms and clinical characteristic of onset and progressive courses with highly pathogenic IAV strains, such as H5N1 and H7N9.

Human airway epithelial lining is an important interface for IAV interactions and therapeutic target of airway diseases. As these cells represent the first physical barrier, they are responsible for the recognition of pathogens with several pattern recognition receptors (PRRs) and orchestrating proinflammatory immune responses [9]. Three major classes of PRRs involved in influenza virus detection are Toll-like receptors (TLRs), retinoic acid inducible gene-I (RIG-I)-like receptors (RLRs) and nucleotide and oligomerization domain, leucine-rich repeat-containing proteins (NLRs) [10]. The NOD-like receptor family pyrin domain containing 3 (NLRP3) inflammasome is the most widely studied NLRP, capable of sensing a range of pathogenic, environmental, and host-derived factors [11]. Following activation, NLRP3 binds to the adaptor protein, an apoptosis-associated speck-like protein containing a CARD (ASC). ASC further recruits the enzyme Caspase-1 to form the inflammasome complex, initiating autocatalytic cleavage of Caspase-1, after which interleukin-1β (IL-1β) and interleukin-18 (IL-18) are matured and secreted [12]. Therefore, exploring the relevance of NLRP3 signal transduction in patients with IAV-induced AECOPD has important clinical and basic research significance.

Currently, the NLRP3 inflammasome is recognized as a major pathway by which the innate immune system recognizes and responds during IAV infections. This pathway may be important in the pathogenesis of severe IAV infection in AECOPD [13]. Therefore, this study aimed to explore the mechanism of airway inflammation and as well as the reasons underlying the quick and aggressive the disease development in AECOPD patients with IAV infections.

## Methods

More detailed methods have been described in the supplementary data.

### Isolation, purification, identification, and culture of human bronchial epithelial cell

#### Isolation of human bronchial epithelial cell

Normal human bronchial epithelial cell (NHBE, *n* = 3) and diseased human bronchial epithelial cell-COPD (DHBE, *n* = 3) harvest was modified from described methods [14, 15]. Briefly, human lung tissues were isolated at the site more than 2 cm distant from the edge of lung cancer (in situ carcinoma) with or without COPD patients. Then transported to the laboratory, tracheas were rinsed with the phosphate-buffered saline (PBS), cut into small segments, and transferred to 1% Protease type XIV and 0.01% Deoxyribonuclease I (DNase I) solution for 8-24 h on platform rocker at 4ºC overnight. Added fetal bovine serum (FBS) to a final concentration of 10% to end dissociation. Collected solutions and distributed into 50 ml tubes. Centrifuged at 500 g for 5 min at 4ºC. Aspirated supernatant and added declumping solution (0.25 mg/ml Collagen Type IV, 2 mM Ethylenediaminetetraacetic acid (EDTA), 0.05 mg/ml Dithiothreitol (DTT), 10ug/ml DNase), incubated for 15-60 min at 37ºC, visually monitoring clump dissociation, added FBS to 10%, centrifuged at 500 g for 5 min. Removed supernatant, and resuspended pellet in DMEM/F12 for counting using a hemocytometer. The study protocols were approved by the ethics committees of both hospitals, and all participants provided written informed consent.

#### Purification, identification, and culture of human bronchial epithelial cell

Bronchial epithelial cells were grown in BEGM medium (Lonza, NJ, USA) overnight on a glass coverslip in 12-well dishes. The medium was removed, and the cells were washed with PBS and fixed in 4% paraformaldehyde for identification. Cells were expanded on dishes coated with type I rat-tail collagen until 70% to 80% were confluent, then detached by trypsin/EDTA at 37ºC for 3-5 min, and collected by centrifugation and resuspended in BEGM for reseeding on collagen-coated 12 mm transwell Clear (Corning Life Sciences, USA) supported membranes at a density of 2.0 × 10^5^/insert. When confluent, the apical medium was withdrawn to create an air–liquid interface. Cells were propagated at 37ºC of 5% CO_2_ for 3 to 4 weeks and medium in the basic compartment changed every 2 to 3 days. The source airway epithelial cells were identified by performing immunofluorescence assays, and immunohistochemical and hematoxylin–eosin staining. All images were obtained using a 5000B Leica microscope equipped with a charge-coupled device camera (Retiga 200R) interfaced with Q-Capture Pro software (Q image). NHBEs and DHBEs were used for infection and treatment at passage two. Influenza virus was diluted in BEGM and added to cells.

#### Influenza A virus production and plaque assay

Seasonal influenza A/Anhui/1/2017 (H3N2) strain was kindly provided by Prof. Yan Liu (Department of Microbiology, Anhui Medical University). Virus aliquots were stored in liquid nitrogen, and freeze/thaw cycles were avoided. IAV/H3N2 virus was propagated in 10-day-old specific pathogen-free embryonated chicken eggs at 35℃ and 55–65% humidity. Allantoic fluid was harvested 48 h post-infection. Then IAV/H3N2 virus was grown in Madin-Darby canine kidney (MDCK) cells. Infectious influenza virus titer was determined by standard plaque assay on MDCK cells in the presence of 2 µg/ml L-1-tosylamido-2-phrnylrthyl chloromethyl ketone (TPCK)-treated trypsin. Confluent monolayers of MDCK cells were infected with stock virus or serially diluted in 1% bovine serum albumin DMEM for 2 h at 37 °C. 3% low-melting agarose in H_2_O by autoclaved at 121ºC for 15 min; cooled, solidified agarose can be melted by the microwave oven and keep at 45ºC incubator. Agarose was mixed with 2 × DMEM at 1;1; v/v, and be removed to the 42ºC water bath until use. Then gently aspirated media out of influenza infected monolayer well, added 2 ml of the agarose/growth media mixture to each well, let the plate sit for 15 min at RT as the agarose overly solidify, moved the plates to incubator for 72 h at 37 °C. The overlay was removed, the cell were fixed with 3.65% formaldehyde at RT for 1 h, the formaldehyde and agarose were discarded. The monolayers were stained for 5 min by 0.5% crystal violet, and plaques were counted.

#### RNA sequencing

Total RNA was isolated from NHBE and DHBE cells using Trizol reagent (Invitrogen). Both cells were infected with IAV (multiplicity of infection (MOI) = 2) for 24 h and analyzed in triplicate. RNA libraries were prepared using the VAHTS®mRNA-seq V2 Library Prep Kit for Illumina from Vazyme and sequenced on an Illumina HiSeq 2000 System (Sangon Biotech, Shanghai, China). Raw RNA-seq sequenced reads were quality tested using FASTQC and were aligned to GRCh38/hg38 Homo sapiens reference genome using STAR aligner with ENCODE standard options for long RNA-seq pipeline. RSEM was used to estimate gene expression abundance and calculate TPM values. Differential gene expression was carried out with DESeq2. Significant differently expressed genes were defined as having and adjusted *P* < 0.05, a log2 change > 1.

#### Western blotting

After being washed with PBS, cells and lung tissues were harvested using RIPA buffer and boiled for 10 min in 100ºC. Total protein was separated on 4–12% SDS–PAGE (Thermo Scientific, USA) and transferred to a PVDF membrane. After blockade with 5% non–fat dry milk in TBST (50 nM Tris–HCl, 150 mM NaCl, 0.05% Tween 20, pH 7.5) for 1 h at room temperature, the membrane was incubated with primary specific antibodies at 4ºC overnight, and subsequently incubated with secondary HRP-conjugated antibody for 4 h at room temperature. An enhanced chemo-luminescence detection kit (ECL Advance, UK) was employed. Densitometry for all proteins was normalized against loading control.

#### Quantitative real-time PCR

RNA was extracted and reversed transcribed to cDNA using high capacity cDNA reverse transcription. Real-time PCR (RT-PCR) was performed using the SYBR Premix Ex Taq II (Tli RNaseH Plus) (Takara Biotechnology, Dalian, China). Expression levels of target mRNA were calculated using 2^−∆∆^Ct relative to the reference gene (β-actin), then calculated as fold change relative to media control.

#### RNA interference

The siRNA oligonucleotides were synthesized by Gene Pharma Company (Shanghai, China). DHBE cells seeded in 12-well plates were transfected with siRNAs using Lipofectamine 3000 (Invitrogen, USA) according to the manufacturer’s instructions. In brief, DHBE cells were seeded in 12-well plates at 4.0 × 10^5^ cells/well. DHBE cells were pre-transfected with 40 nM siNLRP3 or siNC for 24 h and 48 h to measure the level of mRNA and protein, respectively, following infection with IAV(MOI = 2) for 3 h. The medium was removed and replaced with fresh opti-MEM. Next, 2 μl siRNAs were diluted in 98 μl of opti-MEM medium and mixed gently. A total of 5 μl of Lipofectamine 3000 was added in 95 μl opti-MEM medium and incubated for 5 min. Diluted siRNA and Lipofectamine 3000 were combined and mixed gently, then incubated for 20 min at room temperature. The siRNA- Lipofectamine 3000 complexes were added to each well and mixed gently. The siRNA final concentration was 40 nM. The cells were then incubated at 37 °C for 48 h prior to use in experiments. The knockdown efficiency was analyzed by RT-PCR and immunoblot.

#### Cell proliferation assays

To assess the effects of MCC950 (Sigma, USA), DHBE were seeded in triplicate in 96-well plates at 5000 cells/well. The medium was removed and replaced with fresh medium containing MCC950 at various concentrations and examined using an MTT (3-[4,5-dimehyl-2-thiazolyl]-2,5-diphenyl-2H– tetrazolium bromide) assay as described previously [16, 17]. In brief, at different time points, 10 µl MTT (5 mg/ml in PBS) was added to each well, followed by incubation at 37ºC. The culture medium was removed and 100 µl dimethylsulfoxide (DMSO) was added to each well followed by shaking at room temperature for 10 min. The spectrometric absorbance at 490 nm was determined using a microplate reader (Thermo Scientific, Waltham, MA, USA).

#### COPD rat modeling

Male Sprague Dawley rats (200–250 g, 6–8 weeks), obtained from the animal services unit of Anhui Medical University, and were given access to food and water ad libitum. Animals were housed in a specific pathogen-free facility with a controlled environment of twelve-hour light/dark cycles. Rats were randomly selected for COPD modeling with exposure to cigarette smoke, and cigarette smoke exposure was given in a custom-designed passive smoking chamber (70 cm × 40 cm × 60 cm) with a house-directed flow inhalation and smoke-exposure system contained in a laminar flow and smoke-extraction unit. Rats were exposed to smoke from 1 cigarette (tar 12 mg, nicotine of flue gas 1.0 mg, carbon monoxide of flue gas 14 mg. Nanjing Tobacco Industry, China) twice/day, 10 cigarettes/30 min/time, six times/week, for 12 weeks. Non-smoking control rats were exposed to air for the same period.

#### Arterial blood gas analysis and pulmonary function tests

Arterial blood gas analysis and pulmonary function tests were performed immediately after the last exposure. Lung function parameters were assessed using a noninvasive rat pulmonary function measurement system (GYD-003, Noninvasive Rat Lung Function Telemetry Device, Emka, France).

#### Influenza virus infection for animals

On the last day of exposure, rats were anesthetized with thiopental sodium and infected intranasally with 2.5 × 10^3^ plaque forming units (PFUs) of IAV/H3N2 in 100 µl. Control rats were sham-inoculated with saline. We recorded the rats’ body weight every day and observed their behavioral changes. Following inoculation, smoking was discontinued to remove the effects of acute smoke exposure, and rats were sacrificed at day 7 post-infection. MCC950 in 3% DMSO vehicle was administered intranasally in COPD rats at a dose of 10 mg/kg daily beginning on day 1 post-infection until tissue harvest, death, or recovery.

#### Histology analysis

Rats were anesthetized, after which lungs were perfused with PBS and fixed in 4% paraformaldehyde overnight at 4ºC, then processed, embedded in paraffin, sectioned, and stained with hematoxylin and eosin (HE).

#### Bronchoalveolar lavage fluids (BALF)

A 20 − gauge angiocatheter was ligated into the trachea and the lungs were lavaged with sterile saline (5 ml). The lavage fluid specimens were centrifuged at 500 g for 10 min at 4 °C, and the supernatant was stored at − 80 °C for detection.

#### Enzyme-linked immunosorbent assay

The concentrations of IL-1β and IL-18 in serum and BALF supernatant were determined using human and rat enzyme-linked immunosorbent assay (ELISA) kits (CUSABIO Life Sciences, MD, USA) according to the manufacturer’s instructions.

#### Statistical analysis

Statistical analysis was performed using SPSS 23.0 (SPSS Inc., Chicago, IL, USA). The normal distribution of continuous variables was examined using the Kolmogorov–Smirnov test. Normal distribution variables were expressed as mean ± standard deviation (SD), and non-normal variables were reported as median with interquartile range (IQR). The means of skewed variables was compared by Mann–Whitney U test. Student's *t* test and one- or two-way ANOVA were used to compare the means of two and three groups of normally distributed variables. Categorical variables were analyzed by Pearson’s chi-square test. Survival analyses of COPD rats were conducted using Kaplan­Meier analysis and the Cox proportional hazards model. *P* < 0.05 was considered significant.

## Results

### Isolation, purification, identification, and culture of human bronchial epithelial cells

We isolated human bronchial epithelial cells from normal control (NHBE) and COPD patients (DHBE). Cells are characteristic of cobblestone morphology. Daily cell monitoring with optical inverted microscope recorded dynamic growth process at different stages in two weeks (Figure S1A). Cells were identified with epithelial cell-typical antibody cytokeratin 17/19 (Figure S1B and S1C). Isolated epithelial cells have a strong proliferative ability after continuous culture within 5 passages. The successful isolation and culture of human bronchial epithelial cells laid an experimental foundation.

### RNA sequencing of NHBE and DHBE cells after IAV inoculation

**Fig. 1:**
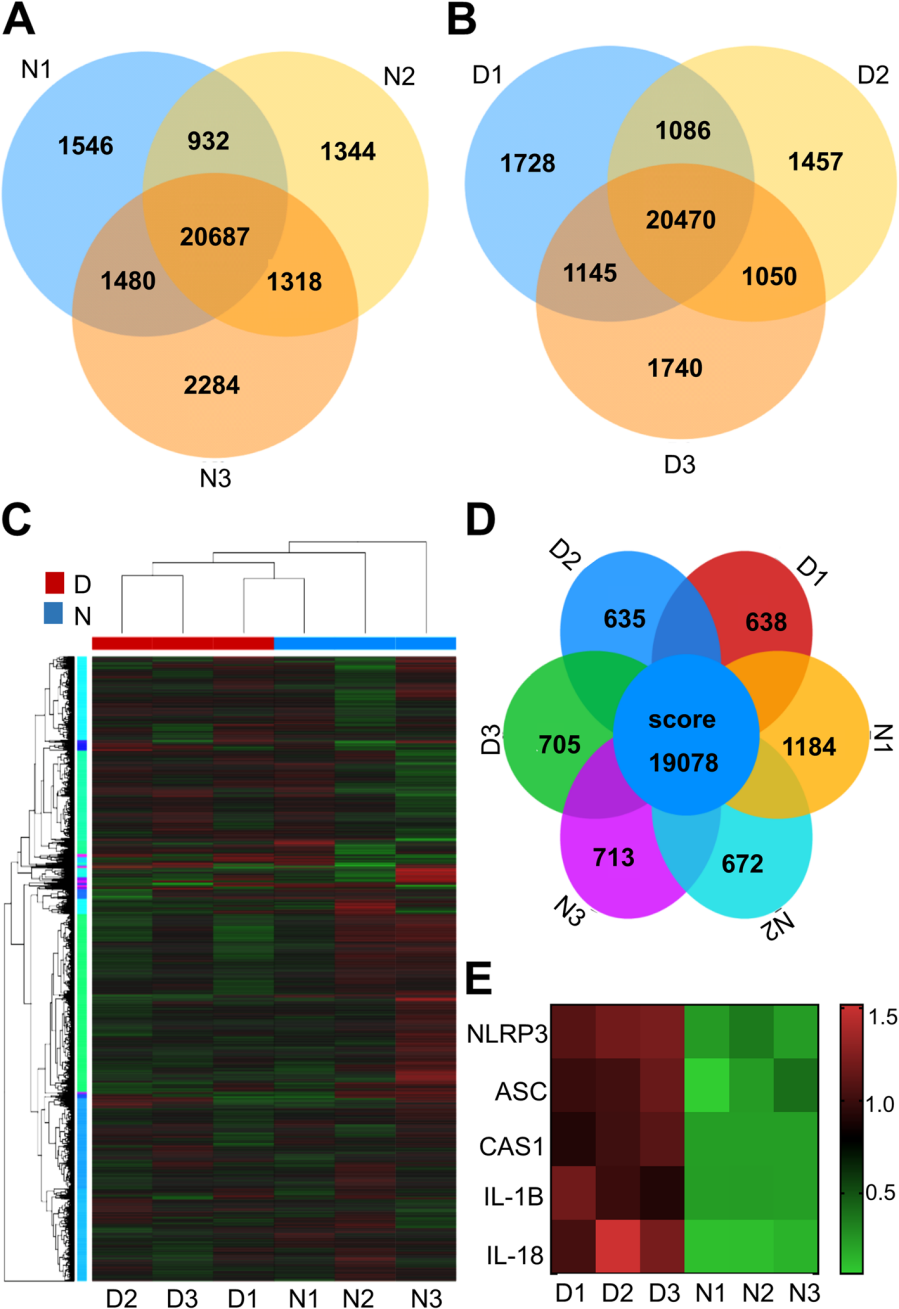
RNA sequencing of NHBE and DHBE cells after inoculation with IAV. **A** The gene expression number of NHBE cells after 24 h incubation with IAV. **B** The gene expression number of DHBE cells after 24 h incubation with IAV. **C** The differentially expressed genes in DHBE cells compared with the NHBE cells are shown in the heat map. **D** The differentially expressed gene numbers in NHBE and DHBE cells. **E** NLRP3 inflammasome pathway differentially expression is shown in the heat map

To explore the occurrence and progression of airway inflammation caused by IAV, we established an air–liquid interface and identified epithelial cells from COPD patients and healthy controls. Subsequently, the gene expression profiles of NHBE and DHBE cells were revealed by RNA sequencing to further explore the possible regulatory mechanism. The two cell groups were infected with IAV (MOI = 2) for 24 h, and this step repeated in triplicate (Figs. 1A & 1B). A total of 19,078 differentially expressed genes were identified (Figs. 1C & 1D), of which 83 were significantly upregulated and 197 were downregulated in the DHBE cells compared to the NHBE cells (Table S1-2). Interestingly, the NLRP3 inflammasome-associated genes were also differentially expressed (Fig. 1E), suggesting that the priming of the NLRP3 inflammasome signaling pathway may be related to the pathogenesis of AECOPD.

### IAV activates and fires airway inflammation in bronchial epithelial cells through the NLRP3 inflammasome pathway

**Fig. 2:**
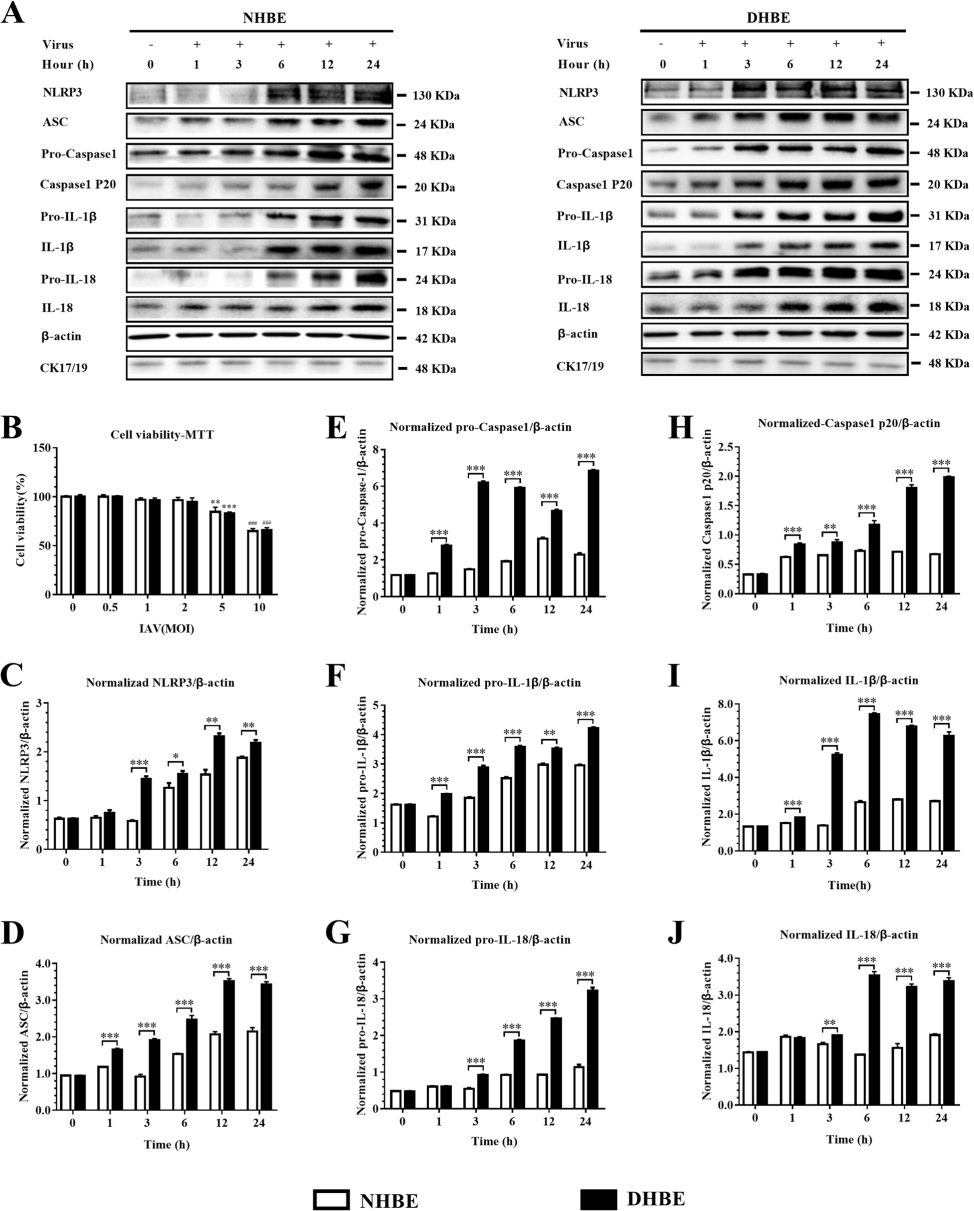
IAV activates and fires airway inflammation in bronchial epithelial cell through NLRP3 inflammasome pathway. **A** NHBE (*n* = 3) and DHBE cells (*n* = 3) were incubated with IAV (MOI = 2) for 0, 1, 3, 6, 12, 24 h, respectively; cell lysates were subjected to immunoblotting with the indicated antibodies. CK17/19 is an indicator of epithelial cells; β-actin was used as a reference protein. **B** After IAV (MOI = 0, 0.5, 1.0, 2.0, 5.0, or 10.0) incubation with NHBE/DHBE cells for 24 h, MTT was used to detect cell viability. The normalized ratio of **C** pro-caspase1, **D** caspase1 p20, **E** NLRP3, **F** pro-IL-1β, **G** IL-1β, **H** ASC, **I** pro-IL-18 and **J** IL-18 expression to β-actin were measured at the above time points (*denote *P* < 0.05, **denotes *P* < 0.01, ***denotes *P* < 0.001). Data are presented as the mean ± SD of three independent experiments

To detect the effect of optimal IAV concentration on cell viability, NHBE and DHBE cells were treated with 0, 0.5, 1.0, 2.0, 5.0, or 10.0 MOI (*P* < 0.01 and *P* < 0.001, respectively; Fig. 2B). We infected NHBE and DHBE cells with IAV (MOI = 2) for 1, 3, 6, 12 or 24 h (Fig. 2A) and measured the expressions of NLRP3 inflammasome components. The levels of NLRP3 inflammasome components in DHBE cells were significantly increase at 3 h after inoculation, reached the highest levels at 12 h, and maintained relatively stable levels for 24 h. In contrast, the NLRP3 inflammasome components in NHBE cells started at 6 h, and increased gradually from 6 to 24 h. In addition, the levels of NLRP3 inflammasome components in DHBE cells were significantly higher than in NHBE cells (Fig. 2C-J).

**Fig. 3:**
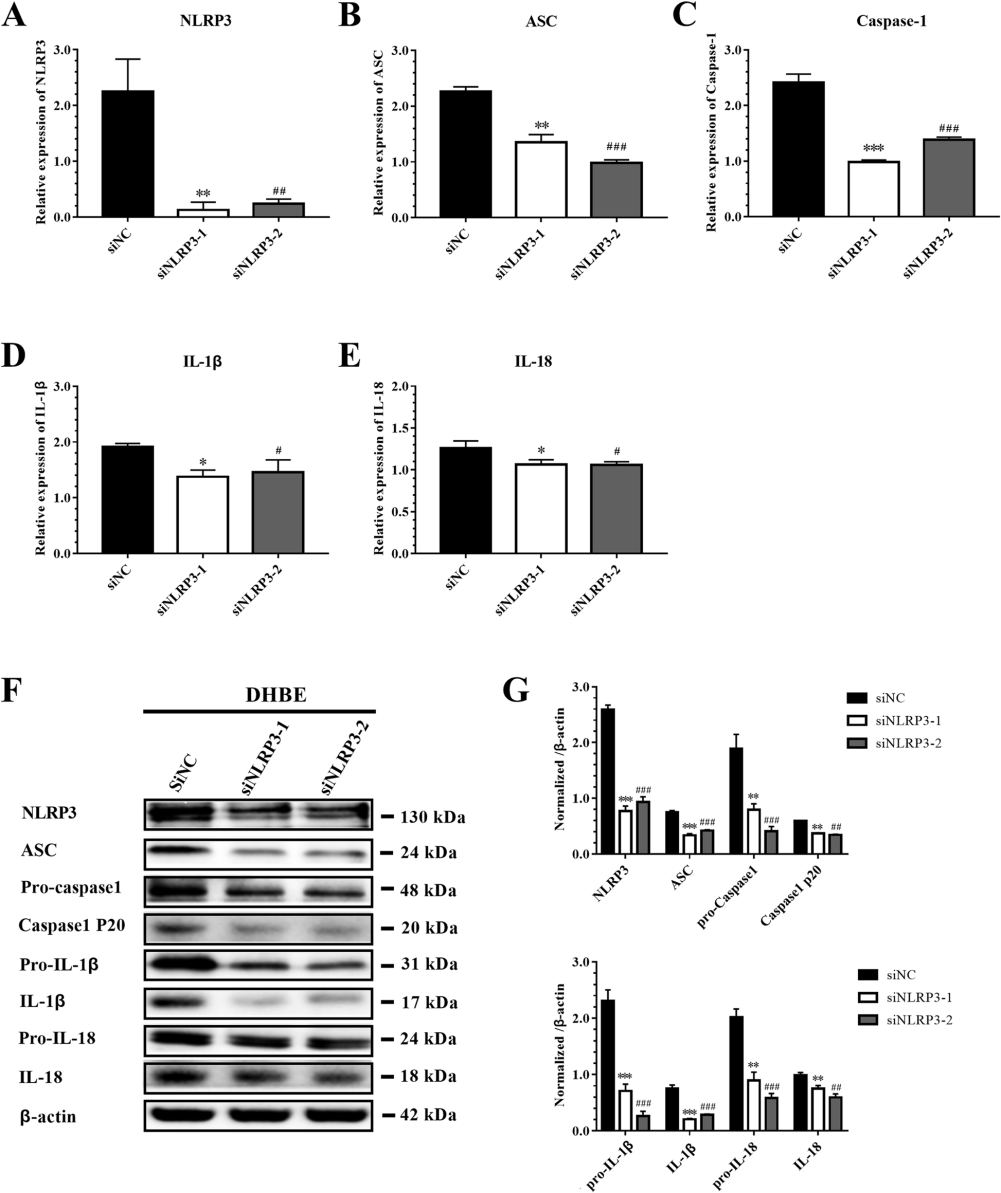
Specific siRNA downregulating NLRP3 inflammasome pathway in DHBE. DHBE cells were pre-transfected with 40 nM siNLRP3 or siNC for 24 h and 48 h for detecting the level of mRNA and protein, respectively, following infected with IAV(MOI = 2) for 3 h. NLRP3 inflammasome components were detected by qRT-PCR.: the levels of **A** NLRP3, **B** ASC, **C** Caspase-1, **D** IL-1β, **E** IL-18 **F** NLRP3 inflammasome components were detected by immunoblot: **G** normalized ratio of NLRP3 inflammation signaling pathway to β-actin. (*denote *P* < 0.05, **denotes *P* < 0.01, ***denotes *P* < 0.001, significant difference between siNC and siNLRP3-1; ^#^denote *P* < 0.05, ^##^denotes *P* < 0.01, ^###^denotes *P* < 0.001, significant difference between siNC and siNLRP3-2). Data are presented as the mean ± SD of three independent experiments

To investigate response of IAV to NLRP3 inflammasome components in epithelial cells, we knocked-down NLRP3 expression through transfection with specific siRNAs (siNLRP3-1 & siNLRP3-2), and designed a negative control (siNC) as the internal control group. Compared to the siNC group, siNLRP3 significantly inhibited NLRP3 mRNA and protein expressions in IAV-infected DHBE cells, and the mRNA and protein levels of ASC, caspase-1, IL-1β and IL-18 were also suppressed when cells were transfected with siNLRP3 (Fig. 3). Consequently, the mRNA and protein levels of ASC, caspase-1, IL-1β, and IL-18 may be dependent on NLRP3 inflammasome activation during the AECOPD.

### MCC950 decreases the expression of NLRP3 inflammasome components in IAV-infected DHBE cells

**Fig. 4:**
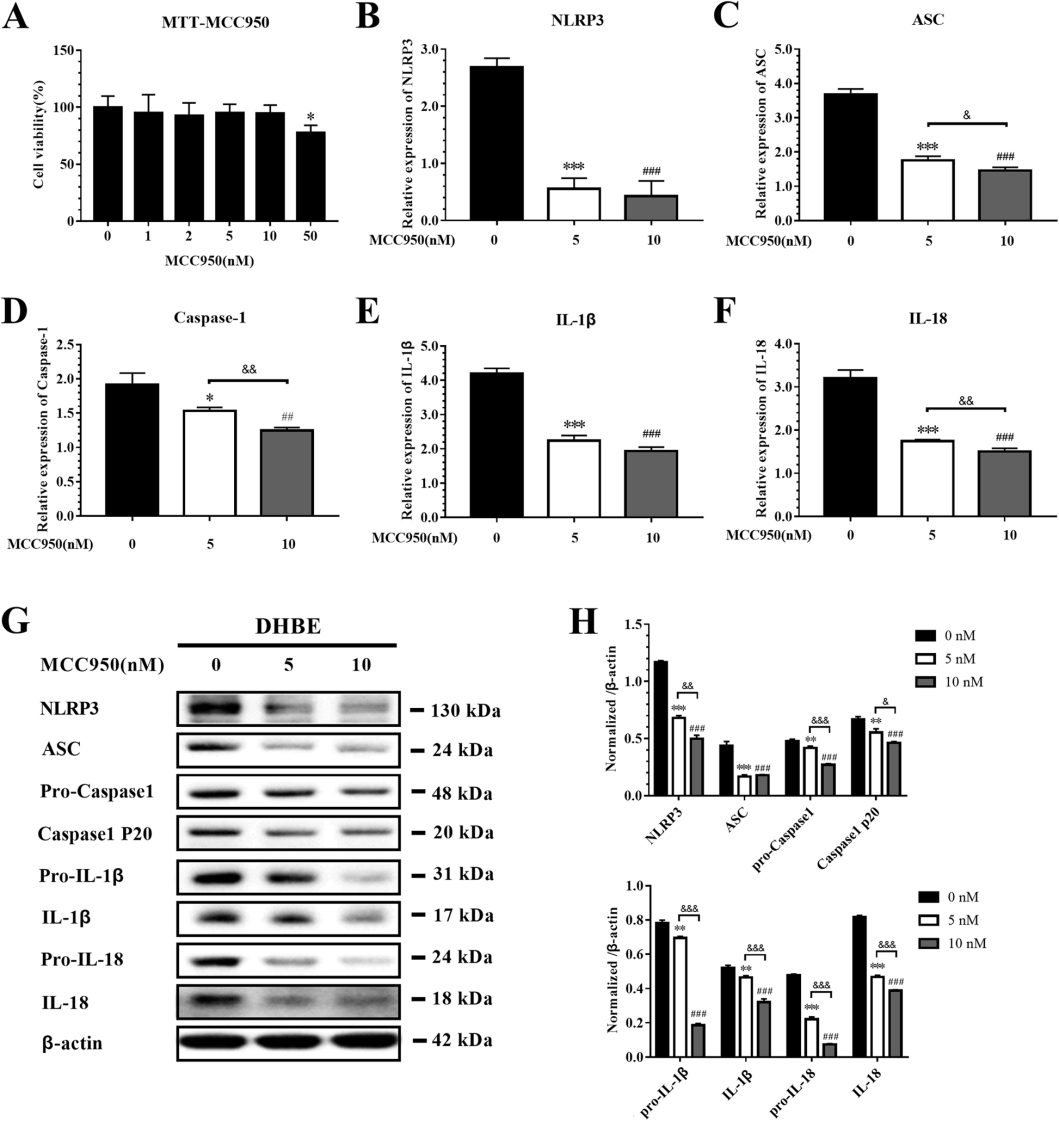
MCC950 inhibits the NLRP3 inflammasome pathway in DHBE. DHBE were infected with IAV at for 24 h (MOI = 2). **A** DHBE cells were treated with different concentrations of MCC950 for 24 h and cell survival was tested by MTT assay. Cells treated with DMSO only acted as control with cell viability set at 100%. The NLRP3 inflammasome components in DHBE were measured by qRT-PCR. **B** NLRP3, **C** ASC, **D** Caspase-1, **E** IL-1β, **F** IL-18 mRNA expression in DHBE. **G** The NLRP3 inflammasome components in DHBE was measured by western blot. **H** Normalized ratio of The NLRP3 inflammasome components to β-actin. (*denote *P* < 0.05, **denotes *P* < 0.01, ***denotes *P* < 0.001, significant difference between 0 and 5 nM; ^#^denote *P* < 0.05, ^##^denotes *P* < 0.01, ^###^denotes *P* < 0.001, significant difference between 0 and 10 nM). Data are presented as the mean ± SD of three independent experiments

To further explore the role of the NLRP3 inflammasome pathway in the pathogenesis of AECOPD, MCC950, an inhibitor of the NLRP3 inflammasome that prevents the activation of caspase-1 and inhibits the maturation and secretion of IL-1β, and IL-18 [18], was used to inhibit the expressions of NLRP3 inflammasome components. DHBE cells treated with MCC950 did not exhibit obvious cytotoxicity in any concentration except 50 nM (Fig. 4A). The mRNA and protein levels of NLRP3, ASC, caspase-1, IL-1β, and IL-18 were significantly decreased in DHBE cells treated with MCC950 during the period of IAV infection, and the inhibitory effects of 10 nM MCC950 were better than those 5 nM MCC950 (Fig. 4B-H). Therefore, these results indicate that NLRP3 inflammasome components have key roles in AECOPD, and that MCC950 can reverse the effects.

### Histopathological changes of rats treated with IAV and MCC950

To further elucidate the above results, we established a COPD rat model. First, lung function parameters were measured in the COPD model group and the normal group. Compared to the normal control, partial pressure O_2_ (PaO_2_), oxyhemoglobin saturation (SaO_2_), special airway resistance (SRaw), air volume per minute (MV), maximum expiratory volume (EV), peak expiratory flow (PEF) and peak inspiratory flow (PIF) were significantly decreased in the COPD model group. In contrast, partial pressure CO_2_ (PaCO_2_) and special airway resistance (SRaw) were significantly increased in the COPD model group (Figure S2), suggesting that the COPD model was successfully established.

**Fig. 5:**
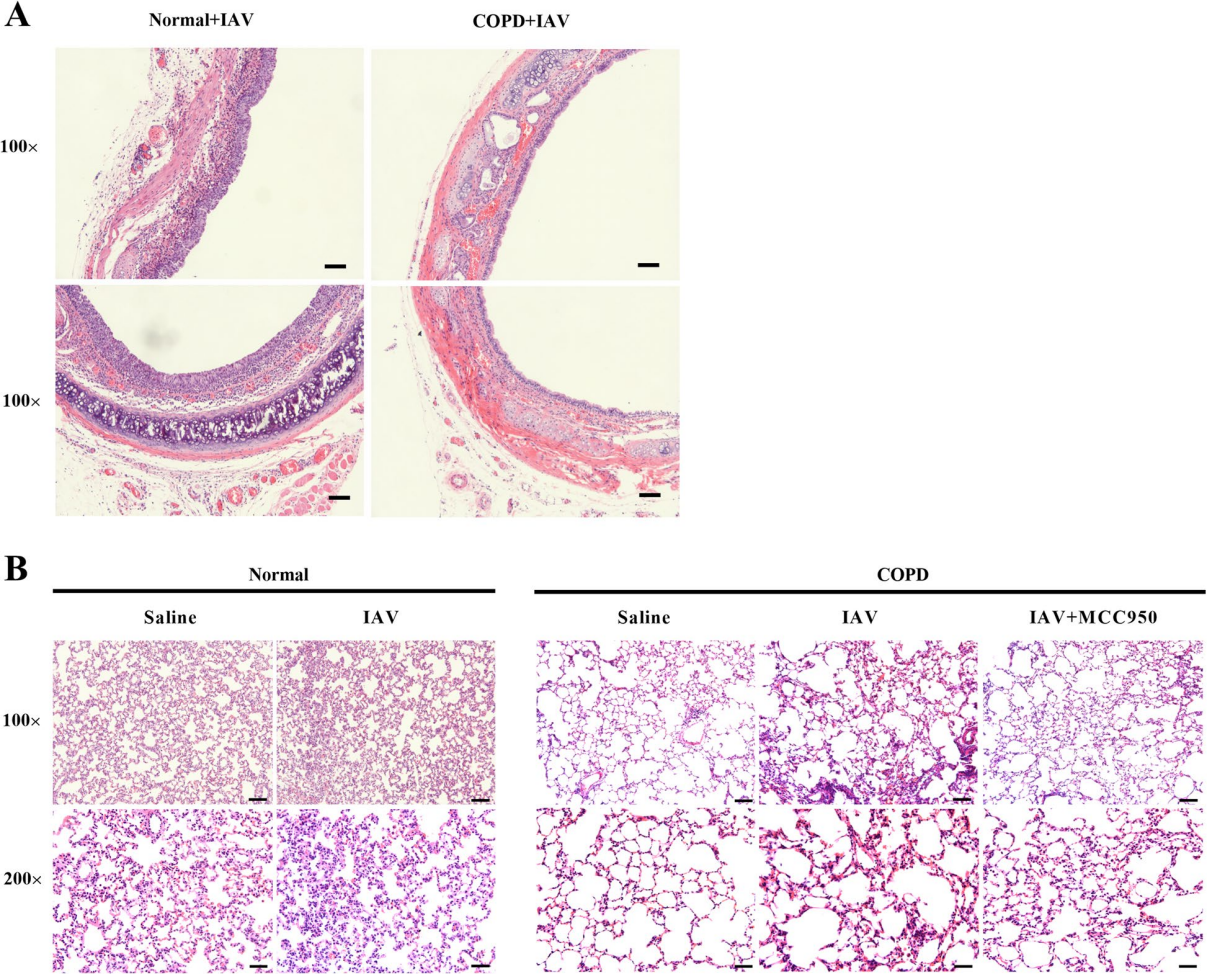
Histopathological changes of rats treated with IAV. After 12 weeks of cigarette-smoke exposure, the airspace was enlarged markedly, the number of alveoli was decreased and the desquamation of bronchiolar epithelial cells was obviously in the lung samples of the rats of the COPD group

Second, we observed the histopathological changes of COPD rats. After 12 weeks of cigarette smoke exposure, the airspace of COPD rats was notably enlarged, the number of alveoli was significantly decreased, and the bronchiolar epithelial cells were obviously desquamated (Fig. 5A). In addition, histological examination on day 7 post-infection demonstrated different degrees of lung injury in COPD rats treated with IAV and MCC950. Compared to saline-treated normal rats, IAV-infected normal rats showed lightly higher interstitial inflammatory infiltration. However, compared to saline-treated COPD rats, IAV-infected COPD rats exhibited extensive lung damage, including desquamation of bronchiolar epithelial cells, damage in diffused alveolar construction, formation of air sac, and severe interstitial inflammation. Moreover, treatment could prevent injuries and inhibit airway inflammatory infiltration in rats (Fig. 5B). These results demonstrated that MCC950 therapy could alleviate IAV-induced lung injury in COPD rats.

### MCC950 improves survival in IAV-induced COPD rats

**Fig. 6:**
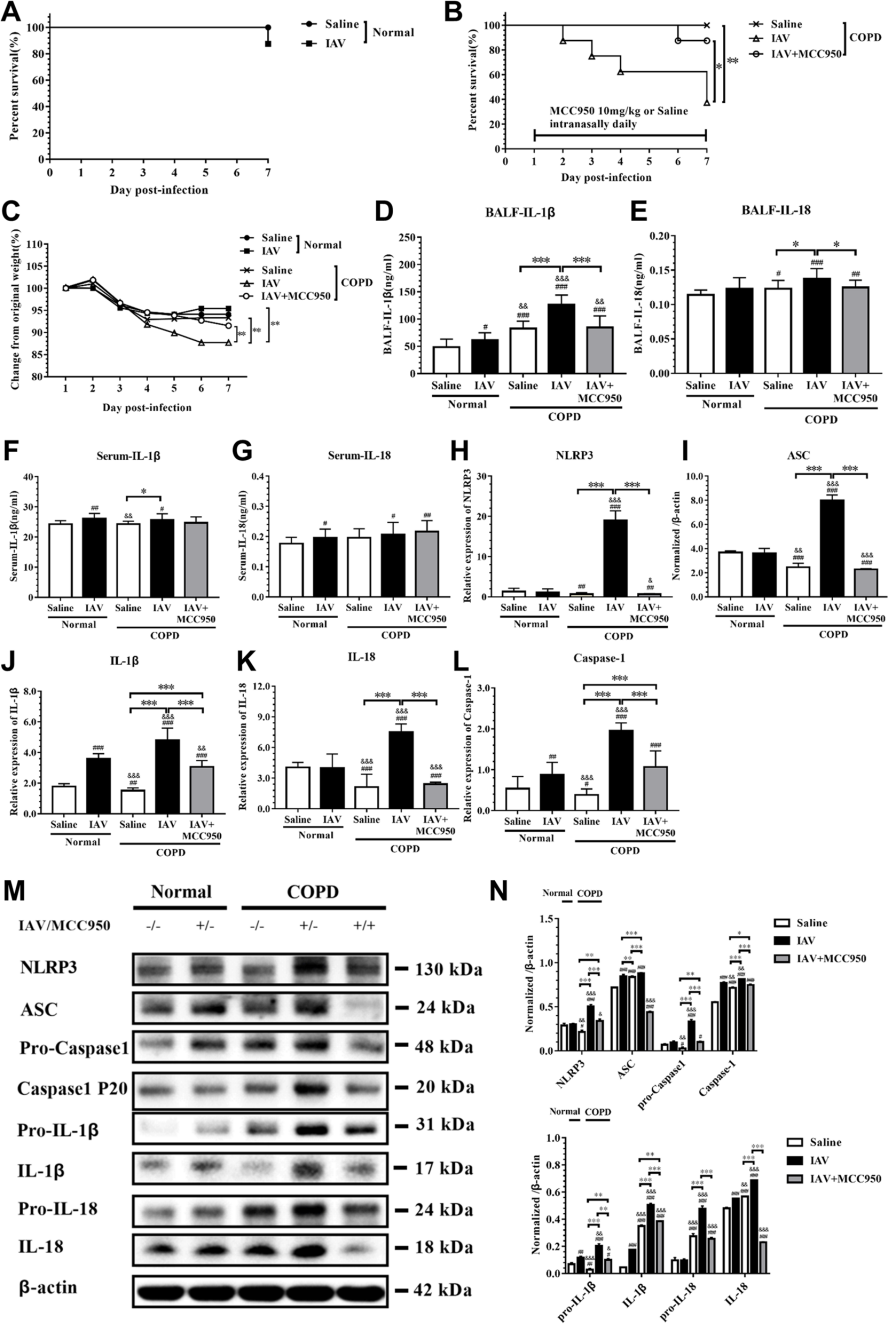
MCC950 inhibits NLRP3 inflammasome pathway in COPD rat model. Rats were infected with IAV (2.5 × 10^3^ PFU intratracheal) and treated with MCC950 (10 mg/kg intranasal daily) or saline control beginning on day 1 post-infection. BALF were collected on 7 days’ post-infection. **A** Mortality of Normal rats, **B** Mortality of COPD rats, **C** Weight loss was recorded; **D** IL-1β and **E** IL-18 in BALF were measured by ELISA. **F** IL-1β and **G** IL-18 in serum were measured by ELISA. The mRNA expression level of **H** NLRP3, **I** ASC, **J** Caspase-1, **K** IL-1β and **L** IL-18 were measured in lung homogenates by qRT-PCR. **M** NLRP3 inflammasome pathway protein expression were measured in lung homogenates by western blot **N** and normalized to β-actin. (*denote *P* < 0.05, **denotes *P* < 0.01, ***denotes *P* < 0.001). Data are presented as the mean ± SD of eight rats per group

We assessed the ability of MCC950 to prevent the activation of NLRP3 inflammasome components. The average survival time of saline-treated normal rats was 7.0 days, with 100% survival rate; the average survival time of IAV-infected normal rats was 7.0 days, with an 87.5% survival rate (Fig. 6A). Additionally, the average survival time of saline-treated COPD rats was 7.0 days, with a 100% survival rate; the average survival time of IAV-infected COPD rats was 5.5 days, with a 37.5% survival rate. Furthermore, MCC950 therapy has a protective effect, as the average survival time was 6.875 days, with an 87.5% survival rate (Fig. 6B). Consistent with the survival results, IAV-infected COPD rats was associated with weight loss (Fig. 6C) and behavioral alterations (e.g., coat ruffling, febrile shaking, and mild lethargy). These findings indicate that IAV reduced the survival time of COPD rats, but MCC950 could reverse this effect.

### MCC950 inhibits the expression of NLRP3 inflammasome components in COPD rats

To explore the role of the NLRP3 inflammasome pathway in the pathogenesis of COPD rats, we examined the expression of NLRP3 inflammasome components in BALF and serum of IAV-infected rats and MCC950-treated rats. The concentrations of IL-1β and IL-18 in BALF were significantly elevated in IAV-infected rats, whereas in MCC950-treated rats, they were attenuated (Figs. 6D and 6E). However, there was no significant difference in serum (Figs. 6F and 6G). In addition, the mRNA levels (Figs. 6H-L) and protein expressions (Figs. 6M and 6N) of NLRP3 inflammasome components were significantly increased in lung homogenates of IAV-infected rats, and the opposite results were observed in MCC950-treated COPD rats. These results are consistent with the previous in vitro findings*.*

### Elevated levels of IL-1β and IL-18 in serum and BALF in AECOPD patients

Experimental research is the foundation, on which diseases are investigated and treatments found; thus, we enrolled AECOPD patients and measured their IL-1β and IL-18 levels in serum and BALF. A total of 37 AECOPD patients are included, and 19 were IAV-negative while 18 were IAV-positive. Their clinical characteristics were shown in Table S3. The FEV_1_/FVC ratio was dramatically declined in the positive group compared to the negative group. The levels of IL-1β and IL-18 in both serum and BALF were significantly higher in the IAV-positive group than the IAV-negative group (Figure S3), suggesting that IAV-positive patients have more severe obstruction and inflammation than IAV-negative patients. In addition, the levels of IL-1β and IL-18 in BALF were higher than the levels in serum, indicating that the inflammatory response in BLAF is more sensitive and rapid than that in serum. Therefore, IAV may be involved in the triggering of COPD exacerbation through the NLRP3 inflammasome pathway.

## Discussion

**Fig. 7:**
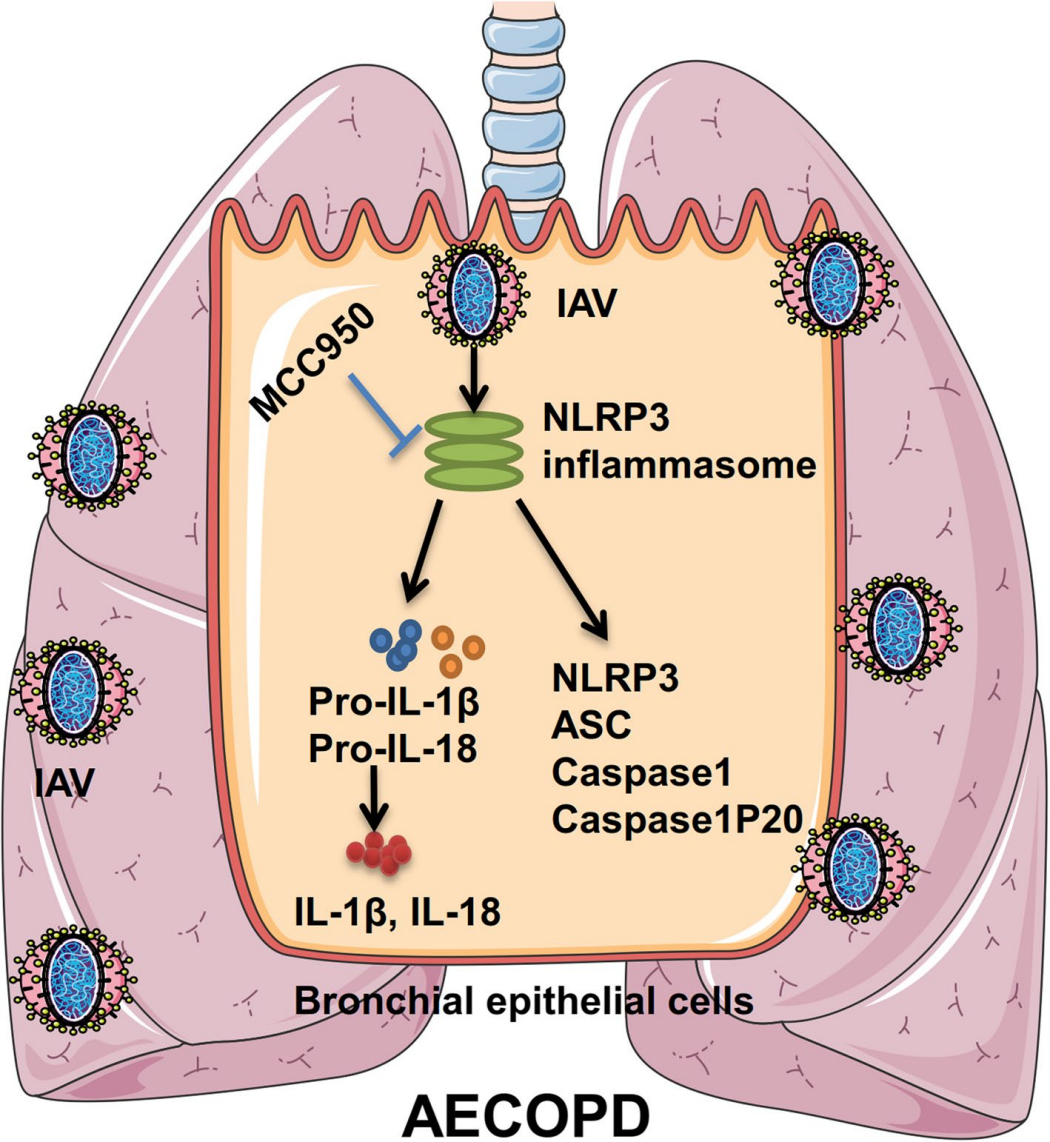
The role of IAV induced NLRP3 inflammasome activation in AECOPD. Invasion of IAV promotes the NLRP3 inflammasome activation in bronchial epithelial cell, which contribute to increase secretion of pro-inflammatory cytokines and triggers acute exacerbation of COPD; whereas MCC950 therapy reverse the progression

There is growing evidence that COPD patients are more susceptible to influenza viral infection, which subsequently leads to more severe symptoms and accelerates declining lung function. However, the cellular and molecular mechanisms of AECOPD are poorly understood. Therefore, current therapeutic strategies have limited efficacy, and new approaches are urgently required. In this study, we explored the underlying mechanisms of IAV-induced airway inflammation by triggering the activation of the NLRP3 inflammasome pathway, conducting experiments both in vitro and in vivo. We found that NLRP3 inflammasome-associated genes were differentially expressed; IAV induced earlier and stronger airway inflammatory storms in DHBE cells and COPD rats by activating the NLRP3 inflammasome pathway, but MCC950 treatment could reverse the effects (e.g., MCC950 improved survival rate and suppressed inflammatory response in AECOPD rats). In addition, we explored the mechanisms by which IAV induced severe illness and rapid progression in AECOPD patients, which may provide strong evidence for a potential treatment for AECOPD patients (Fig. 7).

Bronchial epithelial cells act as a defensive barrier to initiate and orchestrate immune and inflammatory responses by releasing chemokines and cytokines [19]. Therefore, successful isolation and identification of the DHBE and NHBE cells provides an excellent research platform for us to investigate the pathogenesis of AECOPD (Figure S1). According to our data, a total of 19,078 differentially expressed genes were identified between NHBE and DHBE cells (*n* = 3), of which the expression of NLRP3 inflammasome and components and downstream IL-1β and IL-18 correlated with inflammasome action were upregulated consistently (Fig. 1) (Table S1-2). Recent researches recognized the NLRP3 inflammasome as a major pathway by which the innate immune system recognizes and responds during IAV infections [20–22]. We found there was rare researches about the role of NLRP3 inflammasome components in IAV induced COPD exacerbation. Based on those, we proposed hypothesis and found that NLRP3 inflammasome may play an important role in AECOPD. Subsequently, we found that DHBE cells had earlier inflammatory reactions at 3 h, while NHBE cells showed reactions at 6 h, and the expressions of NLRP3 inflammasome components in DHBE cells were significantly elevated (Fig. 2), suggesting that DHBE cells showed more severe inflammatory responses. In addition, we found that siNLRP3 reduced the expression of NLRP3 inflammasome components. These results confirmed that the expressions of IL-1β and IL-18 are dependent on the activation of the NLRP3 inflammasome (Fig. 3). These results indicate that early prevention and effective treatment for AECOPD is essential [23].

The protective role the NLRP3 inflammasome pathway plays during IAV infection has been previously identified by establishing NLRP3-deficient mice [24, 25]. Three integral in vivo studies identified the critical role of the NLRP3 inflammasome in the innate immune antiviral response. Conversely, recent studies have shown that IAV induces excessive production of cytokines by activating NLRP3 activation, leading to lung injury and death [20, 26]. Accumulating evidence emphasizes the role of the NLRP3 inflammasome in mediating the proinflammatory response to IAV infection as well as the importance of the relationship between beneficial and detrimental activation in determining whether infection will resolve or lapse into hyperinflammation. Therefore, our results provide an indication of the role of NLRP3 and identify therapeutic targets and drugs that could dampen NLRP3 responses in chronic inflammatory disease, especially AECOPD.

Pathogen clearance and host survival depend on adequate activation of the innate immune system, but overwhelming inflammatory responses to IAV can be harmful to the host [26]. Therefore, early modulation of NLRP3 inflammasome activity can limit the production of injurious inflammatory cytokines, prevent the death of pyroptotic cells, and prevent the destruction of tissue. MCC950 has been shown to be a specific NLRP3 inhibitor and to be protective in injurious NLRP3 inflammasome activation [21, 27]. In vitro, the treatment of IAV-infected THP-1 macrophages with MCC950 promotes alveolar epithelial cell repair [28]. In vivo, it is effective at doses ranging from 4 to 20 mg/kg in mouse models of autoimmune encephalitis, a disease of constitutive NLRP3 activation [3]. In the present study, to elucidate the underlying mechanisms of IAV-infection outcomes in COPD patients and to identify new therapeutic approaches, we utilized models that showed exaggerated IAV infection in DHBE cells and in COPD rats. The effect of MCC950 against IAV was detected in DHBE cells, and MCC950 treatment decreased the levels of NLRP3 inflammasome components (Fig. 4). In the COPD model, we demonstrated that rats exposed to cigarette smoke for 12 weeks displayed decreased lung function (Figure S2) and increased airway inflammation (Fig. 5). Interestingly, the protection from IAV-induced inflammatory response was significantly associated with a decreased amount of NLRP3 inflammasome components, and the concentrations of IL-1β and IL-18 were decreased in BALF after MCC950 treatment compared to the concentrations in COPD rats with IAV infection (Fig. 6).

We really noticed that there were some previous studies have proved that ASC acted as a control in the inflammasome complex. Other researches have shown that the expression of ASC might change in some pathological situations [22, 29, 30]. For example, Wang et al. [31] found that chronic paraventricular nucleus (PVN) infusion of MCC950 significantly decreased ASC, pro-caspase-1, and IL-1β expression in high-salt diet rats. Xu et al. [32] found that compared with the vehicle-treated group, the protein level of NLRP3, ASC, caspase-1 p20 and IL-1β were significantly decreased in the MCC950-treated group (*p* < 0.05). Thus, it is reasons that the expression of ASC might change duo to the functional interaction with complex adjustment. We need more experimental to prove in the future studies. NLRP3-dependent production of IL-1β and IL-18 bind their cell-surface receptors (IL-1R and IL-18R, respectively) expressed on a range of cell types induce potent NF-κB-dependent secondary cytokine about IAV induced inflammation and COPD exacerbation [33, 34]. Therefore, we hypothesize that the exacerbation of COPD in lung injury may be due to overwhelming inflammatory cytokine storm. These results further confirmed in the COPD rat model that NLRP3 signal transduction was involved in the underlying cellular and molecular mechanisms of airway inflammatory storm, and revealed rapid deterioration of simulated airways in AECOPD patients.

In previous clinical investigations, we found that influenza virus is the most frequent strain involved in AECOPD [6]. Thus, we expanded previous observations to detect outcomes of IAV-infected airway inflammation. In this study, the serum and BALF concentrations of IL-1β and IL-18 in AECOPD patients with IAV infection were increased, especially the BALF specimen (Figure S3), indicating that IAV infection is a trigger factor for AECOPD. Our results were supported by other studies that suggest a close relationship between inflammasome-dependent cytokines and COPD, and the mRNA and cytokine levels of NLRP3 and IL-1β were significantly elevated in bronchial tissues of patients with exacerbated COPD [35, 36]. In addition, our results indicated that the levels of IL-1β and IL-18 were significantly higher in BALF than in serum. The reasonable explanation for this observation is that cellular cytokine recruitment mainly originates from the lung microenvironment to regulate the inflammatory response of pathogens; these findings are consistent with clinical observations of earlier and more severe clinical symptoms in the respiratory system [37]. Furthermore, we found that the FEV_1_/FVC ratio decreased as the severity of obstruction in the IAV-positive group increased, which may be related to the increased levels of cytokines (e.g., IL-1β and IL-18) and involved in the pathogenesis of AECOPD (Table S3). Finally, some limitations should be considered. In our study, COPD was aggravated by respiratory pathogen infection, which may bias the clinical results. Furthermore, because only 37 AECOPD patients were included, further research with a larger sample size is needed to verify current findings.

## Conclusions

IAV infection is the most common factor for AECOPD, and worsens airway inflammatory reactions via activating NLRP3 inflammasome signal transduction. Cascading airway inflammation manifests during the earlier stage, persists for a longer period, and leads to more severe clinical symptoms in COPD patients. This study provides strong evidence for the mechanism by which the NLRP3 inflammasome is involved in the cellular and molecular mechanisms of airway inflammation at different levels of AECOPD, and identifies a potential target to treat this disease.

## Supplementary Information

**Additional file 1: Table S1. Table S2. TableS3. **Subject characteristics. **Figure S1.** Isolation,purification, identification, and culture of human bronchial epithelial cells.** A** The cell growth stateswere observed and photographed under the inverted microscope. The pictures ofcultured cells were taken on day 1, 4, 7, 14, respectively. (Scale bar = 200 µm, originalmagnification: ×100; scale bar = 100 µm, originalmagnification: ×200).** B** The isolated NHBE and DHBE cells were cultured for 14 days, then probed with anti-human CK17/19antibody. Immunofluorescence of positive CK17/19 staining (green), DAPI stainedfor nuclear content (blue) (scale bar = 100 µm, originalmagnification: ×200). **C **HE and immunocytochemistry of positive CK17/19 staining (scale bar=100 µm, originalmagnification: ×200). **Figure S2. **Characteristics of COPD rat model. Rats were exposed to cigarette smoke or normal air for 12weeks and then infected with IAV on the last day of exposure. Blood gas analysis and pulmonary function test were conducted. **A** Partial pressure O_2_(PaO_2_) and Partial pressure CO_2_(PaCO_2_) **B** Oxyhemoglobin saturation (SaO_2_) **C **Special airwayresistance (SRaw) **D** Special airwayconductivity (SGaw) **E **Air volume per minute (MV) **F** Maximumexpiratory volume (EV) **G **Peakexpiratory flow (PEF)** H** Peak inspiratory flow (PIF).** I** HE-stained lung sections from rats 7 days’post-infection with 2.5×10^3^ PFU of IAV and treatment with 10 mg/kg MCC950 or salinecontrol. Images shown are representative of 8 rats for each condition,Scale bars, 200 µm. Data are presented as the mean ± SD of eight rats per group. **FigureS3. **Increased levels of IL-1β and IL-18 in serum and BALF in AECOPD patients. The levels of IL-1β and IL-18 in serum and BALF were significantelevated in IAV positive group compare to IAV negative group. (* denote* P*<0.05,*** denotes *P*<0.001).

## Data Availability

The datasets used and/or analyzed during the current study are available from the corresponding author on reasonable request.

## Abbreviations

COPD: Chronic obstructive pulmonary disease
AECOPD: Acute exacerbation of chronic obstructive pulmonary disease
IAV: Influenza A virus
PRRs: Pattern recognition receptors
TLRs: Toll-like receptors
RLRs: Retinoic acid inducible gene-I (RIG-I)-like receptors
NLRs: Nucleotide and oligomerization domain, leucine-rich repeat-containing proteins
NLRP3: NOD-like receptor family pyrin domain containing 3
ASC: Apoptosis-associated speck-like protein containing a CARD
IL-1β: Interleukin-1β
IL-18: Interleukin-18
NHBE: Normal human bronchial epithelial cell
DHBE: Diseased human bronchial epithelial cell-COPD
MDCK: Madin-Darby canine kidney
MOI: Multiple of infection
FPKM: Fragments per kilobase of exon per million reads mapped
PFUs: Plaque forming units
BALF: Bronchoalveolar lavage fluids
