# Supplementary material for: Influenza a virus triggers acute exacerbation of chronic obstructive pulmonary disease by increasing proinflammatory cytokines secretion via NLRP3 inflammasome activation
Source: J Inflamm (Lond). 2022 Jun 23;19:8. doi: 10.1186/s12950-022-00305-y (PMC9219228; doi:10.1186/s12950-022-00305-y)
Supplement: Supplementary file 1 — Additional file 1: Table S1. Table S2. TableS3. Subject characteristics. Figure S1. Isolation,purification, identification, and culture of human bronchial epithelial cells. A The cell growth stateswere observed and photographed under the inverted microscope. The pictures ofcultured cells were taken on day 1, 4, 7, 14, respectively. (Scale bar = 200 µm, originalmagnification: ×100; scale bar = 100 µm, originalmagnification: ×200). B The isolated NHBE and DHBE cells were cultured for 14 days, then probed with anti-human CK17/19antibody. Immunofluorescence of positive CK17/19 staining (green), DAPI stainedfor nuclear content (blue) (scale bar = 100 µm, originalmagnification: ×200). C HE and immunocytochemistry of positive CK17/19 staining (scale bar=100 µm, originalmagnification: ×200). Figure S2. Characteristics of COPD rat model. Rats were exposed to cigarette smoke or normal air for 12weeks and then infected with IAV on the last day of exposure. Blood gas analysis and pulmonary function test were conducted. A Partial pressure O2(PaO2) and Partial pressure CO2(PaCO2) B Oxyhemoglobin saturation (SaO2) C Special airwayresistance (SRaw) D Special airwayconductivity (SGaw) E Air volume per minute (MV) F Maximumexpiratory volume (EV) G Peakexpiratory flow (PEF) H Peak inspiratory flow (PIF). I HE-stained lung sections from rats 7 days’post-infection with 2.5×10^3^ PFU of IAV and treatment with 10 mg/kg MCC950 or salinecontrol. Images shown are representative of 8 rats for each condition,Scale bars, 200 µm. Data are presented as the mean ± SD of eight rats per group. FigureS3. Increased levels of IL-1β and IL-18 in serum and BALF in AECOPD patients. The levels of IL-1β and IL-18 in serum and BALF were significantelevated in IAV positive group compare to IAV negative group. (* denote P<0.05,*** denotes P<0.001). [file 12950_2022_305_MOESM1_ESM.doc]

**Supplementary materials and methods**

**Western blotting**

Anti-NLRP3 (Cell Signaling Technology, USA), anti-ASC (Santacruz Biotechnology, USA), anti-pro- Caspase1 (Cell Signaling Technology, USA), anti-Caspase1 P20 (Cell Signaling Technology, USA), anti-pro-IL-1β (Cell Signaling Technology, USA), anti-IL-1β (Cell Signaling Technology, USA), anti-pro-IL-18 (R&D Systems, USA), anti-IL-18 (R&D Systems, USA), anti-CK17/19 (Cell Signaling Technology, USA) and anti-β-actin (Cell Signaling Technology, USA).

**Primers of quantitative real-time PCR**

The following primers were used: NLRP3 (human) were forward: 5’–GGCAAATTCGAAAAGG GGTATT–3’, reverse:5’–CTGATTTGCTGAGAGATCTTGC–3’; NLRP3 (rat) were forward: 5’–GAG CTGGACCTCAGTGACAATGC–3’, reverse: 5’–ACCAATGCGAGATCCTGACAACAC–3’; ASC (human) were forward: 5’–CTGACGGATGAGCAGTACCA–3’, reverse: 5’–AGTCCTTGCAGGTCC AGTTC–3’; ASC (rat) were forward: 5’–TGGTTTGCTGGATGCTCTGTATGG–3’, reverse: 5’–ACA AGTTCTTGCAGGTCAGGTTCC–3’;Caspase-1(human) were forward:5’–GAAGAAACACTCTGA GCAAGTC–3’, reverse:5’–GATGATGATCACCTTCGGTTTG–3’; Caspase-1 (rat) were forward: 5’–ATGGCCGACAAGGTCCTGAGG–3’, reverse: 5’–GTGACATGATCGCACAGGTCTCG–3’; IL-1β (human) were forward: 5’–GCCAGTGAAATGATGGCTTATT–3’, reverse: 5’–AGGAGCACT TCATCTGTTTAGG–3’; IL-1β (rat) were forward: 5’–AGCTTCCAGGATGAGGACCC–3’, reverse: 5’–GCTCACATGGGTCAGACAGC–3’; IL-18 (human) were forward: 5’–GCTGAAGATGATGAAA ACCTGG–3’, reverse: 5’–CAAATAGAGGCCGATTTCCTTG–3’; IL-18 (rat) were forward: 5’–TGAT ATCGACCGAACAGCCAACG–3’, reverse: 5’–GGTCACAGCCAGTCCTCTTACTTC–3’; β-actin (human) were forward: 5’–CCTGGCACCCAGCACAAT–3’, reverse: 5’–GGGCCGGACTCGTCATA C–3’; β-actin (rat) were forward: 5’–TGTCACCAACTGGGACGATA–3’, reverse: 5’–GGGGTGTTG AAGGTCTCAAA–3’.

**RNA interference**

The following siRNA target sequences were used: Negative control (NC): 5’–UUCUCCGAACG UGUCACGUTT–3’(forward), 5’–ACGUGACACGUUCGGAGAATT–3’ (reverse); si-NLRP3-1: 5’– GCUGCUGAAUGGAUUGAATT–3’(forward),5’–UUCAAUCCAUUUCAGCAGCTT–3’ (reverse); siNLRP3-2:5’–GUGCGUUAGAAACACUUCATT–3’(forward), 5’–UGAAGUGUUUCUAACGCAC TT–3’(reverse).

**ELISA**

Briefly, 96-well fat-bottomed high binding ELISA plates were coated with anti-human or rat IL-1β and IL-18. Samples and standards were added in plates at 37 ºC for 2 h. Plates were then washed (PBS-T, PBS, 0.05 % Tween-20), coated with biotinylated antibody, washed (PBS-T), and avidin-HRP conjugate added. Plated were again washed. Colorimetric reactions were developed by incubation with tetramethylbenzidine (100 µl, 1 mg/ml at room temperature for approximately 5-10 min) and reactions were terminated with sulfuric acid (50 µl; 1M). Run the microplate reader (Thermo Scientific, Waltham, MA, USA) and conduct measurement at 450 nm immediately, calculated by interpolation with the standard curve.

**Subject recruitment and pulmonary function test**

Healthy controls and COPD patients were enrolled in the first affiliated hospital of Anhui Medical University. COPD patient was defined as an established COPD history with a post-bronchodilator forced expiratory volume in 1 second (FEV_1_) to forced vital capacity (FVC) ratio<70% at diagnosis. AECOPD patients were defined as an acute event characterized by a worsening of respiratory symptoms (dyspnea, sputum purulence, or sputum volume) that is beyond normal day-to-day variations, and lead to a change in medication. Patients with other immune-inflammatory diseases such as asthma and bronchiectasis and patients who had received antibiotic or corticosteroids during the past 4 weeks were excluded. Standardized pulmonary function test was performed with a dry spirometer device (Erich Jaeger GmbH, Hoechberg, Germany) at spirometer minutes after inhaling salbutamol 400 µg according to standardization. Clinical characteristics and the FEV_1_, FVC, and FEV_1_/FVC ratio were recorded.

**Nasopharyngeal swab and serum collection**

The nasopharyngeal swab was rotated five to six times and allowed to remain for 5 seconds, then immediately immersed in 3 ml viral preservation liquid (Youkang Technology Company, Beijing, China). Serum of these participants was collected for further analysis.

**Bronchoalveolar lavage fluids (BALF) of human**

BALF was performed using flexible electronic bronchoscope (Olympus, Japan) after local anesthesia with lidocaine. 50 ml sterile saline was instilled into the right middle lobe or the left lingular segment of the lung. BALF was retrieved by gentle vacuum suction of 50 to 100 mmHg，the usual retrieved portion was>30%，and put into sterile containers.

**Virologic assays**

The nasopharyngeal swab preservation liquid and BALF were centrifuged for 500 g for 10 min, then total nucleic acids were extracted and purified using the mini kit (QIAamp MinElute Virus Spin Kit, Hilden, Germany). Analyses included influenza A (H1 and H3), influenza B, respiratory syncytial virus, human metapneumovirus, parainfluenza virus types 1, 2, 3 and 4, coronaviruses (CoV OC43, CoV 229E, CoV NL-63, and HKU1), enterovirus/rhinovirus, adenovirus, and human Boca virus by a Respiratory Vital Panel FAST v2 kit which is based on the multiplex-PCR (polymerase Chain reaction), xMAP (Flexible Multi-Analyte Profiling) and xTAG techniques (Luminex, Toronto, Canada). All samples virologic assays were performed at the department of the clinical laboratory in the first affiliated hospital of Anhui Medical University.

**Bacteriological assays**

Sputum was obtained in a sterile container prior to the initiation of AECOPD treatment and examined within 30 minutes as described previously.

**Supplementary results**

**Table S**3. Subject characteristics

| Variable | IAV  negative | IAV  positive | *p*  value |
| --- | --- | --- | --- |
| Subjects, n | 19 | 18 | NA |
| Male sex, n (%) | 13(68.42) | 13(72.22) | NA |
| Age-years, mean (SEM) | 70 (2.35) | 69 (2.82) | 0.90 |
| BMI (kg/m^2^) ^a^, medium (IR) | 20.98  (18.10-26.01) | 21.35  (19.82-23.61) | 0.16 |
| FEV_1_(L), medium (IR) | 1.12  (0.67-1.41) | 0.82  (0.64-1.27) | 0.49 |
| FEV_1_ (%) of predicted， medium (IR) | 54.60  (28.2-63.30) | 40.55  (27.05-64.78) | 0.77 |
| FEV_1_/FVC ratio ^b^, mean (SEM) | 59.52 (1.88) | 53.29 (1.76) | 0.009 |
| Cigarette, pack-years, medium (IR) | 6 (0-20) | 15 (0-20) | 0.59 |

**Abbreviations:** NA=not applicable; **^a^**BMI=body mass index; ^b^FEV_1_=forced expiratory volume in 1 second; FVC=forced vital capacity.


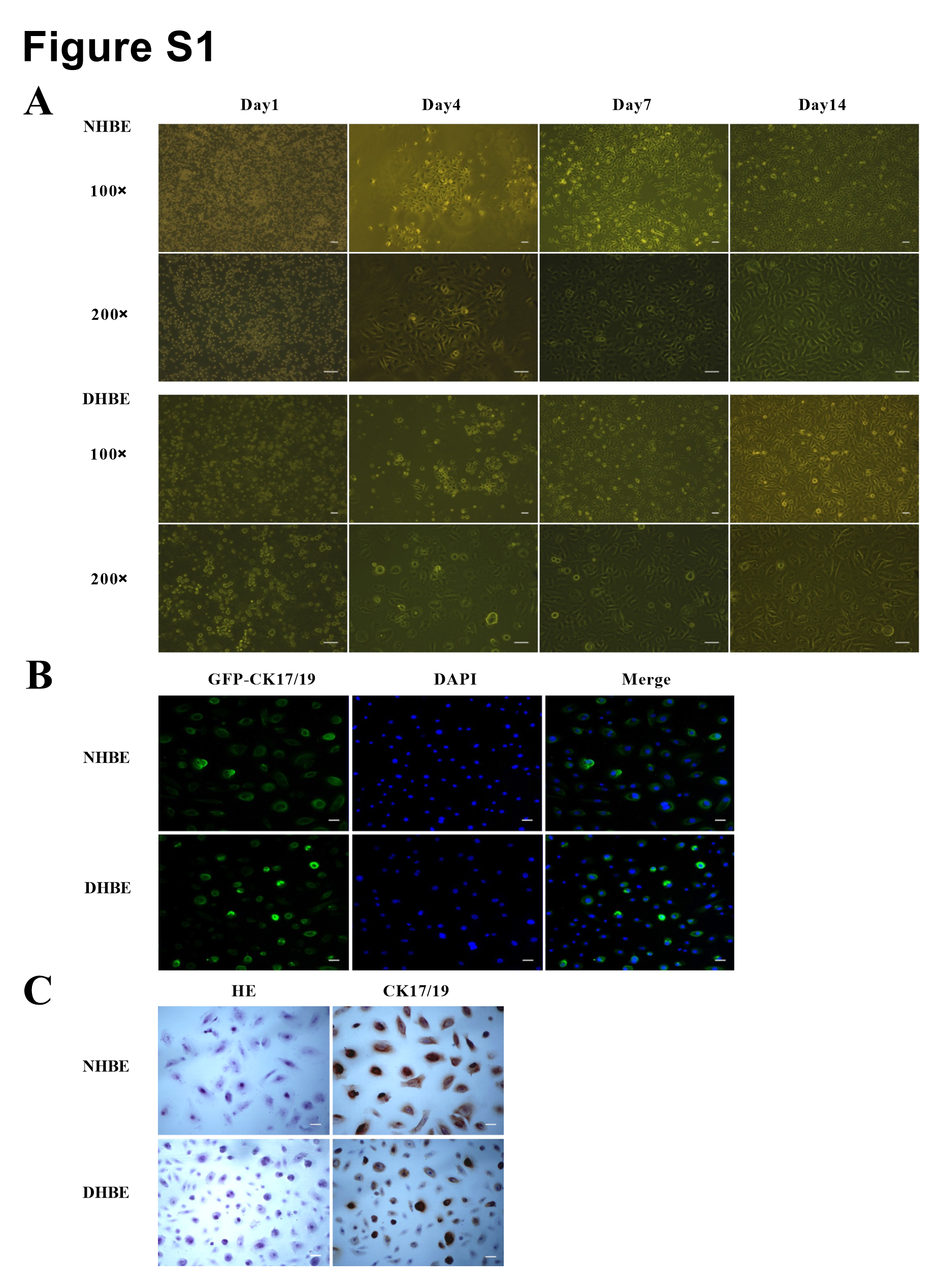


**Figure S1.** **Isolation, purification, identification, and culture of human bronchial epithelial cells. (A)** The cell growth states were observed and photographed under the inverted microscope. The pictures of cultured cells were taken on day 1, 4, 7, 14, respectively. (Scale bar = 200 µm, original magnification: ×100; scale bar = 100 µm, original magnification: ×200). **(B)** The isolated NHBE and DHBE cells were cultured for 14 days, then probed with anti-human CK17/19 antibody. Immunofluorescence of positive CK17/19 staining (green), DAPI stained for nuclear content (blue) (scale bar = 100 µm, original magnification: ×200). **(C)** HE and immunocytochemistry of positive CK17/19 staining (scale bar=100 µm, original magnification: ×200).


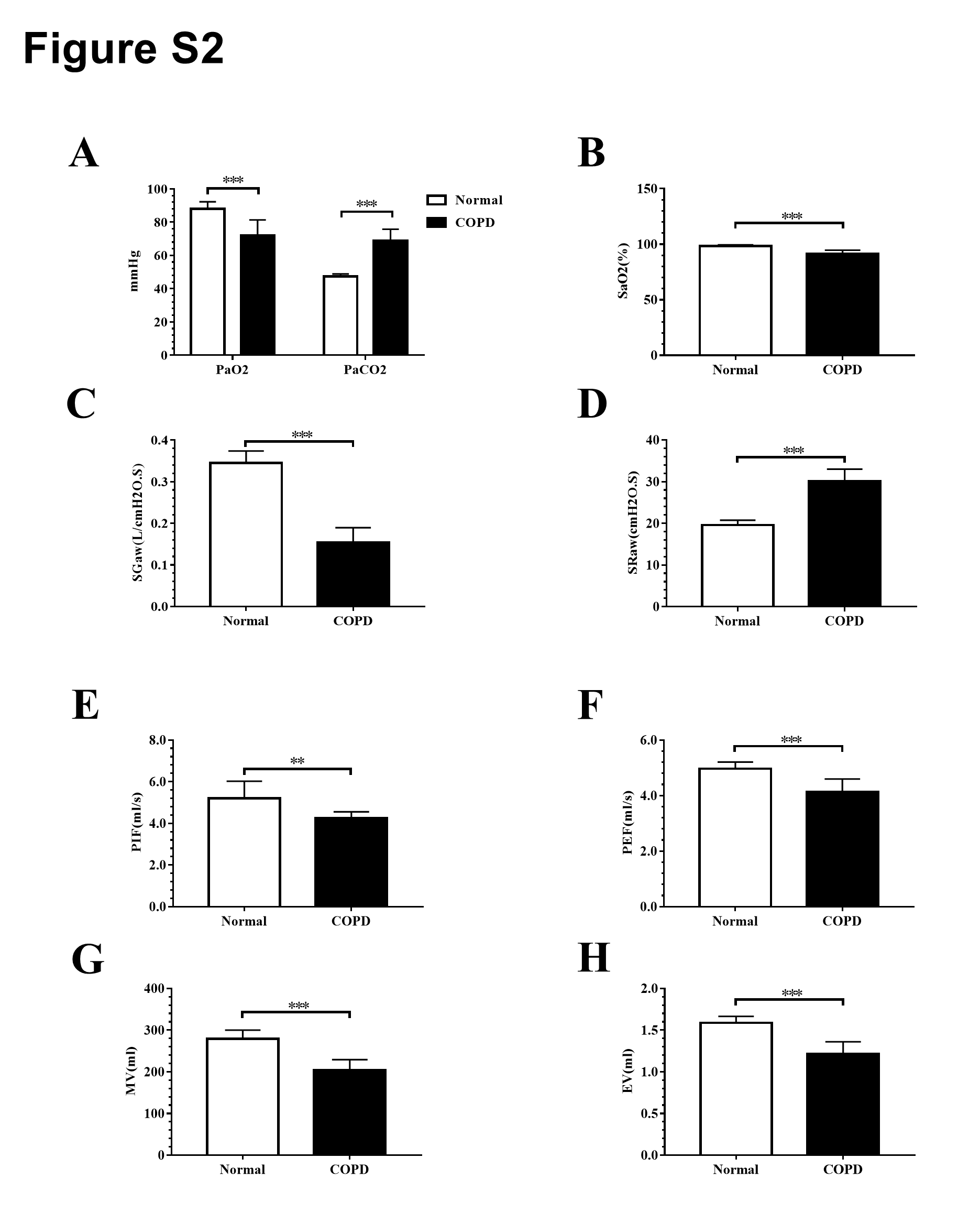


**Figure S2. Characteristics of COPD rat model.** Rats were exposed to cigarette smoke or normal air for 12 weeks and then infected with IAV on the last day of exposure. Blood gas analysis and pulmonary function test were conducted. **(A)** Partial pressure O_2_ (PaO_2_) and Partial pressure CO_2_ (PaCO_2_). **(B)** Oxyhemoglobin saturation (SaO_2_); **(C)** Special airway resistance (SRaw) **(D)** Special airway conductivity (SGaw) **(E)** Air volume per minute (MV) **(F)** Maximum expiratory volume (EV) **(G)** Peak expiratory flow (PEF) **(H)** Peak inspiratory flow (PIF). **(I)** HE-stained lung sections from rats 7 days’ post-infection with 2.5×10^3^ PFU of IAV and treatment with 10 mg/kg MCC950 or saline control. Images shown are representative of 8 rats for each condition, Scale bars, 200 µm. Data are presented as the mean ± SD of eight rats per group.


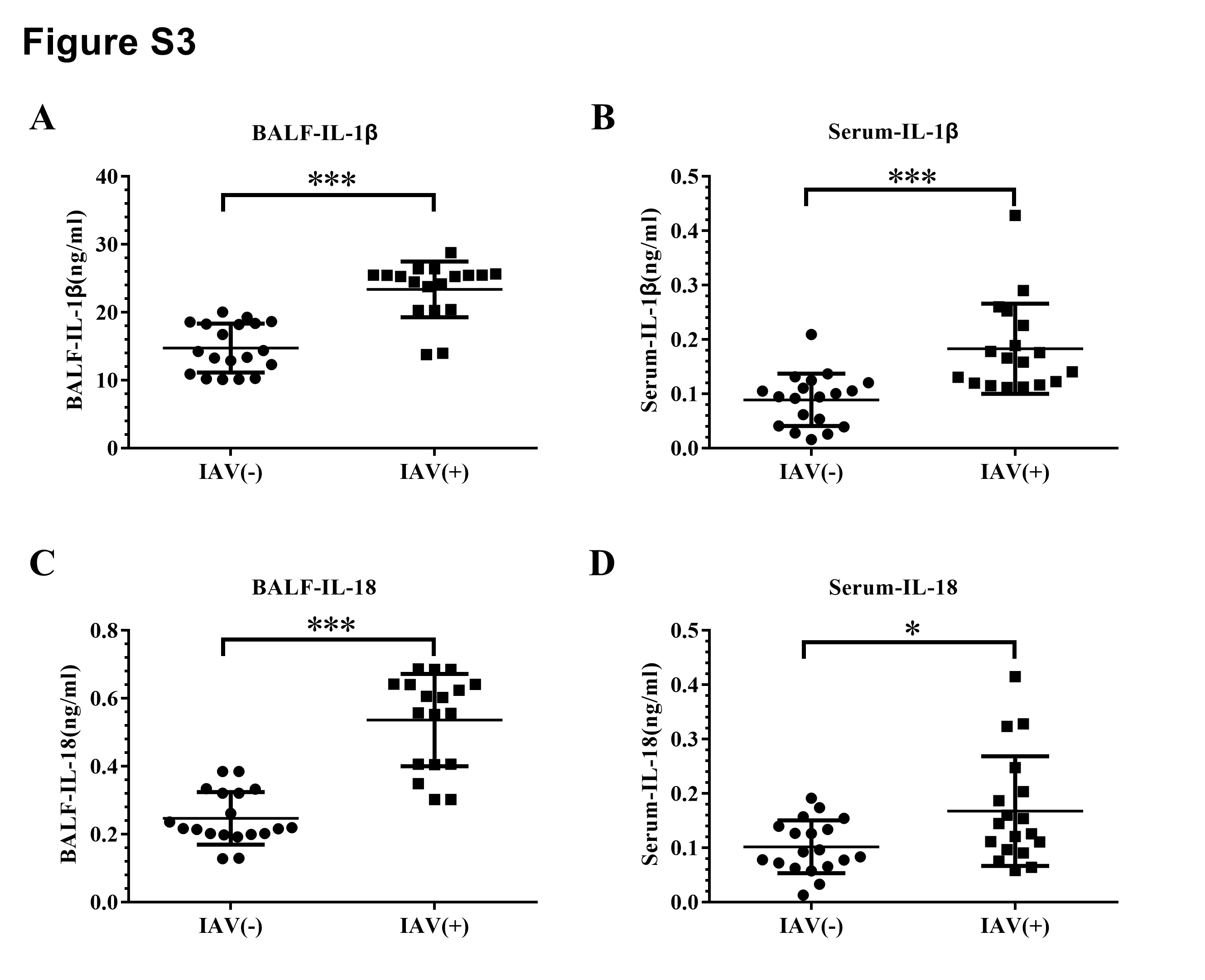


**Figure S3.** I**ncreased levels of IL-1β and IL-18 in serum and BALF in AECOPD patients.** The levels of IL-1β and IL-18 in serum and BALF were significant elevated in IAV positive group compare to IAV negative group. (* denote *P*<0.05, *** denotes *P*<0.001).
